# Heart Rate Variability Analysis in an Experimental Model of Hemorrhagic Shock and Resuscitation in Pigs

**DOI:** 10.1371/journal.pone.0134387

**Published:** 2015-08-06

**Authors:** Edgard Salomão, Denise Aya Otsuki, Andre Luis Correa, Denise Tabacchi Fantoni, Fernando dos Santos, Maria Claudia Irigoyen, Jose Otavio Costa Auler

**Affiliations:** 1 LIM08-Laboratory of Anesthesiology, Department of Anesthesia and Surgical Intensive Care, Faculdade de Medicina da Universidade de São Paulo, São Paulo, Brazil; 2 Hypertension Unit, Heart Institute (InCor), Faculdade de Medicina da Universidade de São Paulo, São Paulo, Brazil; German Red Cross Blood Service Frankfurt, GERMANY

## Abstract

**Background:**

The analysis of heart rate variability (HRV) has been shown as a promising non-invasive technique for assessing the cardiac autonomic modulation in trauma. The aim of this study was to evaluate HRV during hemorrhagic shock and fluid resuscitation, comparing to traditional hemodynamic and metabolic parameters.

**Methods:**

Twenty anesthetized and mechanically ventilated pigs were submitted to hemorrhagic shock (60% of estimated blood volume) and evaluated for 60 minutes without fluid replacement. Surviving animals were treated with Ringer solution and evaluated for an additional period of 180 minutes. HRV metrics (time and frequency domain) as well as hemodynamic and metabolic parameters were evaluated in survivors and non-survivors animals.

**Results:**

Seven of the 20 animals died during hemorrhage and initial fluid resuscitation. All animals presented an increase in time-domain HRV measures during haemorrhage and fluid resuscitation restored baseline values. Although not significantly, normalized low-frequency and LF/HF ratio decreased during early stages of haemorrhage, recovering baseline values later during hemorrhagic shock, and increased after fluid resuscitation. Non-surviving animals presented significantly lower mean arterial pressure (43±7vs57±9 mmHg, P<0.05) and cardiac index (1.7±0.2vs2.6±0.5 L/min/m^2^, P<0.05), and higher levels of plasma lactate (7.2±2.4vs3.7±1.4 mmol/L, P<0.05), base excess (-6.8±3.3vs-2.3±2.8 mmol/L, P<0.05) and potassium (5.3±0.6vs4.2±0.3 mmol/L, P<0.05) at 30 minutes after hemorrhagic shock compared with surviving animals.

**Conclusions:**

The HRV increased early during hemorrhage but none of the evaluated HRV metrics was able to discriminate survivors from non-survivors during hemorrhagic shock. Moreover, metabolic and hemodynamic variables were more reliable to reflect hemorrhagic shock severity than HRV metrics.

## Introduction

Hemorrhagic shock is responsible for high mortality rates in civilian injuries (34%) and combat casualties (90%)[1,2]. The initial care of these patients comprehends an early assessment of hypovolemia, bleeding management and fluid resuscitation [3]. Patient monitoring by traditional vital signs as heart rate, arterial pressure and arterial oxygen saturation can be insufficient to an early and accurate diagnosis of hemorrhage severity and shock [4].

The compensatory response to traumatic hemorrhage is driven by the autonomic nervous system [5]. Studies in animal models involving direct recording of neural activity during hemorrhagic shock demonstrated an increased sympathetic activity in response to the initial blood loss. With progression of hemorrhage, a reduction in neural activity is observed as a sign of irreversible shock [6,7]. However, the technique of direct measurement of the sympathetic nerve tone is invasive and, therefore, its use as a clinical tool is limited.

Alternatively, heart rate variability (HRV) metrics are suggested for indirect assessment of cardiovascular autonomic modulation [8,9]. The HRV represents the time differences between successive beat-to-beat intervals and can be evaluate by a number of methods. Time domain analysis is based on analysis of measurements of the normal-to-normal (NN) intervals or the instantaneous heart rate in a continuous electrocardiogram (EKG) record. Frequency domain analysis in another widely used approach of HRV and utilizes spectral methods to interpret the RR tachogram, generating three main frequency components: very low frequency, low frequency and high frequency power components [8]. The low frequency (LF) component derived from the analysis of RR interval is influenced by both the sympathetic and parasympathetic activities. In contrast, the high frequency (HF) is influenced only by the parasympathetic activity. The LF/HF ratio has been proposed as an index of the sympathovagal balance [9].

The correlation between measures of HRV and the volemic state [10–12], ICU mortality [13] and trauma triage/outcome [5] suggests its potential use as a clinical tool [14]. The objective of this study was to compare HRV to traditional hemodynamic and metabolic parameters during hemorrhagic shock and fluid resuscitation. Our hypothesis was that HRV metrics could be an early predictor of mortality, distinguishing survivors from non-survivors.

## Materials and Methods

The study protocol was approved by the Institutional Ethics and Animal Investigation Committee (Comissão de Ética para Analise de Projetos de Pesquisa do HCFMUSP–CAPPesq n.090/11) and was performed in accordance with the Guide for Care and Use of Laboratory Animals [15].

### Animal preparation

Twenty Landrace and Largewhite crossbreed pigs (25.0 ± 2.5 kg) were fasted for 12 hours with free access to water.

Animals were sedated with midazolam (0.25 mg.kg^-1^) and ketamine (5 mg.kg^-1^) intramuscularly. Anesthesia was induced with intravenous propofol (5 mg.kg^-1^) and maintained with isoflurane (1.4% end-tidal concentration) in oxygen (40%) after orotracheal intubation. The lungs were mechanically ventilated with volume-controlled ventilation at a tidal volume of 8 ml/kg and PEEP of 5 cmH_2_O, and the respiratory rate was adjusted to maintain an end-tidal CO_2_ of 40 ± 5 mmHg (Primus; Dräger, Lübeck, Germany). Lactated Ringer’s solution was administered at a rate of 5 mL/kg/h during the entire procedure. Body temperature was maintained between 37°C and 38°C with the use of a heated mat (Medi-therm II, Gaymar Industries, Orchard Park, NY, USA).

After local anesthesia (3 mL lidocaine 2% at each incision site), a pulmonary artery catheter (744H-7.5F; Baxter Healthcare Corporation, Irvine, USA) was introduced through peripheral cut-downs into the right jugular vein, and an arterial catheter was introduced into the right femoral artery for monitoring and blood sample collection. The right femoral artery and vein were also catheterized for blood withdrawal and fluid infusion.

### Monitoring

Heart rate (HR), mean arterial pressure (MAP), central venous pressure (CVP) and mean pulmonary arterial pressure (mPAP) were obtained directly from a multiparametric monitor (IntelliVue MP50, Phillips, Böblinger, Germany). Cardiac output (CO) measurements were obtained by thermodilution (Vigilance II, Baxter Healthcare Corporation). Body surface area (BSA) was calculated according to the formula (k.BW^2/3^; k = 0.09, BW = body weight).

Arterial and mixed venous blood samples were collected simultaneously and immediately analyzed (ABL 555, Radiometer, Copenhagen).

Cardiac index (CI), systemic vascular resistance index (SVRI), pulmonary vascular resistance index (PVRI) and stroke volume index (SVI), oxygen delivery index (DO_2_I), oxygen consumption index (VO_2_I) and extraction ratio (O_2_ER) were calculated utilizing conventional formulae.

Additionally, arterial-venous CO_2_ gradient (CO_2_ gap) was also calculated.

### Heart rate variability analysis

The EKG of all animals was continuously recorded at 1000 Hz to a personal computer using a data acquisition system (MP100 and AcqKnowledge 3.9, Biopac Systems, Santa Barbara, USA). The EKG analysis was performed off-line at eight time points: at baseline; at 5 min (Hemorrhage5), 10 min (Hemorrhage10) and 15 min (Hemorrhage15) during hemorrhage; at 30 min (Shock30) and 60 min (Shock60) after completion of hemorrhage; immediately after completion of resuscitation (R0); and at 1 h (R60), 2 h (R120) and 3 h (R180) after completion of resuscitation. For each time point, 5 min observation periods were analyzed and R-R intervals (RRI) were generated. All data sets were manually analyzed to ensure RRI data was free of ectopy. The overall variability of the R-R interval was assessed in the time and frequency domains by spectral estimation as described elsewhere [16].

Variance, standard deviation of the R-R intervals (SDNN), and square root of the mean squared differences of successive R-R intervals (RMSSD) were analyzed in time-domain methods. The frequency-domain metrics included very low frequency (VLF:0.0–0.02Hz), low frequency (LF:0.02–0.09Hz), high frequency (HF:0.09–2.0Hz) band powers, and the LF/HF power ratio. These frequency bands were based on a study using pharmacological autonomic blockade with atropine and propranolol in pigs [17]. The LF and HF components are expressed in absolute values (ms^2^) and normalized units to represent the relative contribution of each power component to the total variance (power) in the recording.

### Experimental protocol

**Fig 1:**
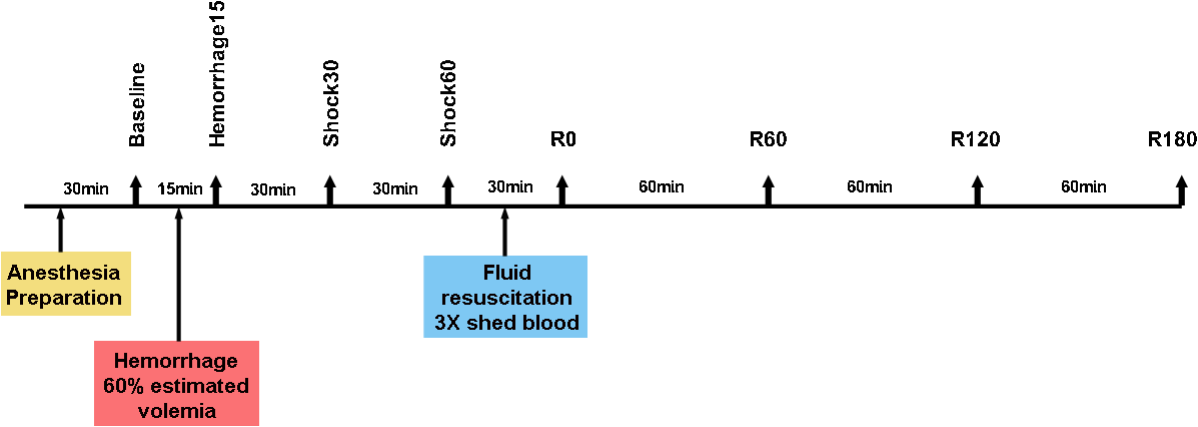
Experimental protocol. Baseline: before hemorrhage; Hemorrhage15: at the end of blood withdrawal; Shock30: 30 minutes after the end of blood withdrawal; Shock60: 60 minutes after the end of blood withdrawal; R0: at the end of fluid resuscitation; R60: 60 minutes after the end of resuscitation; R120: 120 minutes after the end of resuscitation; R180: 180 minutes after the end of resuscitation.

Thirty minutes after preparation, animals were submitted to acute hemorrhagic shock by removing 60% of the estimated blood volume in 15 min (3 mL/kg/min). After hemorrhage, animals were observed for 60 min without treatment. Fluid resuscitation was performed with lactated Ringer´s solution (LR), in a volume of 3 times the volume of blood withdrawn. Animals were observed for 3 hours and those which survived were sacrificed with high concentrations of isoflurane, followed by an intravenous injection of potassium chloride. (Fig 1)

### Statistical analysis

Animals were grouped as surviving hemorrhage / treatment (S group) and non-surviving animals (NS group). All data are presented as mean ± SD or median with interquartile ranges in case of non-normal distribution assessed by the Kolmogorov-Smirnov test. Data were analyzed within group by repeated-measures analysis of variance or Friedman test, followed where appropriate by Tukey or Dunn´s test. Data of surviving and non-surviving animals were compared at baseline, CH0, CH30 and CH60 time points by unpaired t test or Mann-Whitney U test. A multivariable logistic regression analysis with HRV metrics, hemodynamic and metabolic variables was performed to estimate predictive factor for mortality resulting from hemorrhagic shock. A p < 0.05 was considered statistically significant. The GraphPad Prism 5 (GraphPad Software Inc, La Jolla, USA) was used for statistical analysis.

## Results

Seven animals died during hemorrhage and at the first 10 minutes of fluid resuscitation. None of the measured parameters were significantly different between S and NS groups at baseline. No differences were observed in body weight (S: 24.7 ± 2.3 kg; NS: 25.6 ± 3.0 kg, P = 0.45) and in shed blood volume (S: 1,036 ± 97 mL; NS: 1,134 ± 128 mL, P = 0.691) between groups.

### HRV metrics

**Fig 2:**
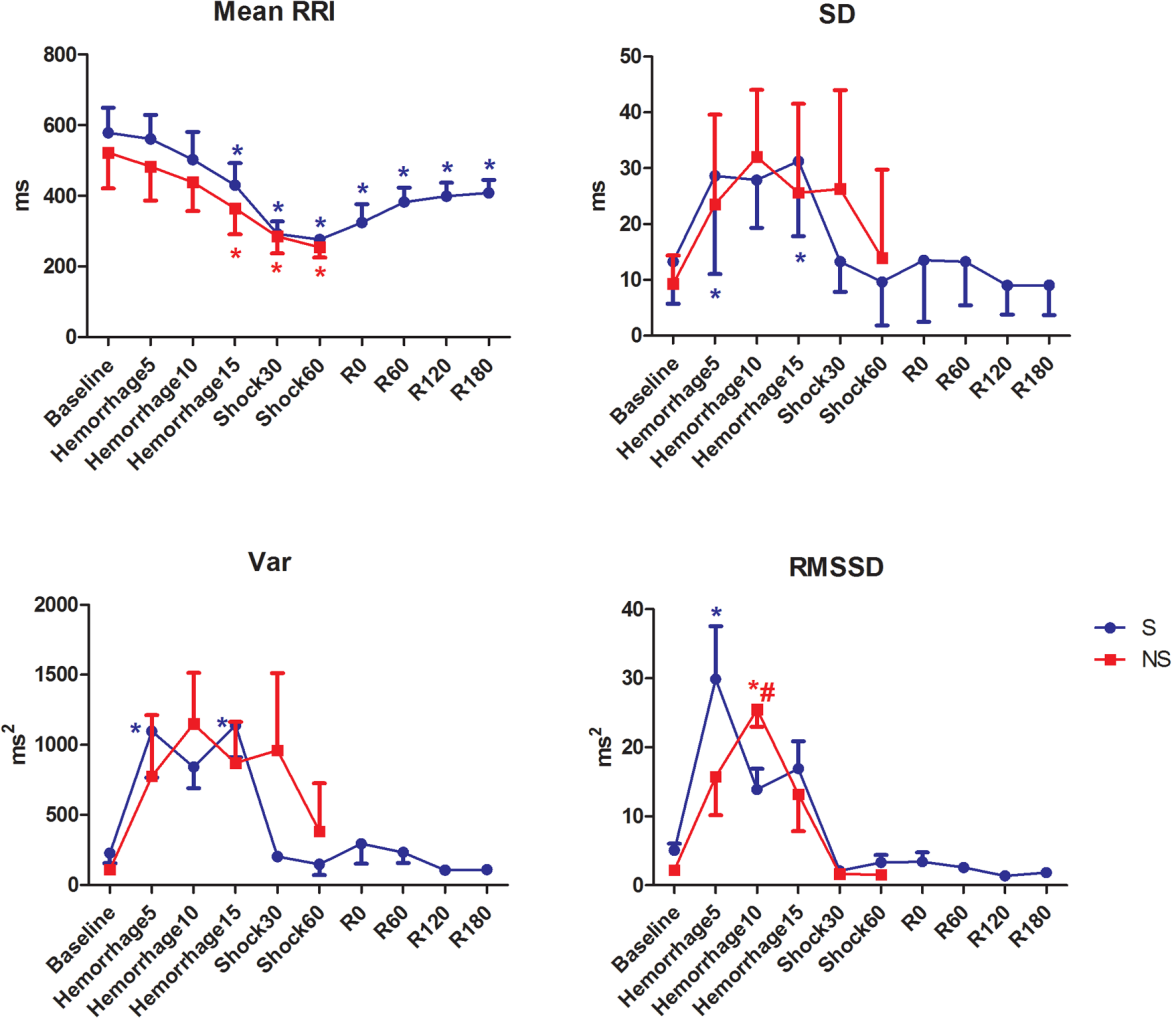
HRV time domain metrics in pigs submitted to acute hemorrhagic shock and fluid resuscitation. S = survivor group. NS = non-survivor group; * = P<0.05 different from baseline; # = p<0.05 NS group different from the S group (t test).

Changes in HRV measurements during hemorrhage and fluid resuscitation are provided in Table 1 and Fig 2. There were no differences in baseline measurements between groups.

**Table 1:** HRV frequency-domain metrics during acute hemorrhagic shock and fluid resuscitation. Mean ± SD.

Variables	Group	Baseline	Hemo5	Hemo10	Hemo15	Shock30	Shock60	R0	R60	R120	R180
VLF abs	S	3,11 ± 3,48	**8,46 ± 9,38** *	1,13 ± 1,08	2,72 ± 2,47	0,94 ± 1,46	0,82 ± 1,18	0,53 ± 0,4256	1,35 ± 1,08	2,17 ± 2,36	2,10 ± 2,15
(ms^2^)	NS	2,40 ± 3,63	6,02 ± 6,09	**22,73 ± 32,07** ^**A**^	457,7 ± 1168	1,54 ± 2,62	0,53 ± 0,61				
LF abs	S	5,06 ± 5,93	12,44 ± 19,77	**21,8 ± 15,43** *	2,23 ± 2,42	1,87 ± 3,98	1,88 ± 3,38	0,84 ± 0,92	2,06 ± 1,69	2,98 ± 2,29	3,35 ± 2,90
(ms^2^)	NS	5,25 ± 5,76	13,69 ± 17,05	54,25 ± 74,29	851,0 ± 2176	3,25 ± 5,76	1,32 ± 1,89				
HF abs	S	5,95 ± 5,94	52,67 ± 144,1	19,12 ± 43,95	56,54 ± 93,15	0,74 ± 1,07	1,20 ± 1,47	0,73 ± 0,57	1,91 ± 0,54	1,14 ± 0,59	1,33 ± 0,79
(ms^2^)	NS	2,99 ± 3,10	3309 ± 8044	**386,6 ± 754,3** ^**A**^	52,67 ± 144,1	1,14 ± 1,04	0,78 ± 0,88				
VLF %	S	16,30 ± 7,56	24,9 ± 13,72	21,8 ± 15,43	26 ± 12,5	24,1 ± 3,93	17,8 ± 5,27	20,82 ± 9,57	23,36 ± 6,93	25,55 ± 7,06	24,91 ± 6,49
	NS	15,14 ± 5,70	25 ± 14,12	14,57 ± 6,27	21,29 ± 19,7	**17,86 ± 5,43** ^**A**^	13,25 ± 7,18				
LF %	S	28,3 ± 17,19	20,0 ± 12,1	21,6 ± 12,76	22,8 ± 13,46	34,2 ± 16,07	32,8 ± 14,77	30,55 ± 11,4	38,09 ± 12,23	**42,0 ± 14,76** **†**	**43,09 ± 13,35** **§**
	NS	36,43 ± 14,11	29,71 ±11,47	19,71 ± 18,74	22,0 ± 10,34	36,86 ± 11,89	30,5 ± 19,43				
HF %	S	55,3 ± 24,15	54,9 ± 24,73	56,4 ± 27,31	51,2 ± 21,38	41,9 ± 15,67	49,5 ± 18,45	48,73 ± 15,21	38,64 ± 14,71	32,55 ± 15,26	32,2 ± 14,26
	NS	48,57 ± 19,28	45,29 ± 18,92	65,71 ± 24,5	56,71 ± 25,71	45 ± 16,41	56,5 ± 25,96				
LF nu	S	37,2 ± 25,75	31,9 ± 23,35	32,51 ± 23,51	36,3 ± 21,48	47,4 ± 20,61	42,7 ± 20,03	41,18 ± 15,43	52,19 ± 16,41	58,27 ± 19,87	59 ± 18,01
	NS	45,14 ± 19,41	43,57 ± 17,8	26,14 ± 24,48	33 ± 20,55	48,29 ± 16,88	37,75 ± 25,14				
HF nu	S	62,8 ± 25,75	68,1 ± 23,35	67,2 ± 23,51	63,7 ± 21,48	52,6 ± 20,61	57,3 ± 20,03	58,82 ±1 5,43	47,82 ± 16,41	41,73 ± 19,87	41,0 ± 18,01
	NS	54,86 ± 19,41	56,43 ± 17,8	73,86 ± 24,48	67,0 ± 20,55	51,71 ± 16,88	62,25 ± 25,14				
LF/HF	S	2,93 ± 4,86	1,65 ± 2,40	1,10 ± 1,22	1,63 ±2,11	2,21 ± 2,24	1,70 ± 1,69	1,25 ± 0,71	2,37 ± 1,93	3,18 ± 2,50	2,97 ± 1,90
	NS	1,74 ± 1,47	1,466 ± 1,18	0,83 ± 1,12	1,08±1,01	2,29 ± 2,12	1,87 ± 1,92				

VLF = spectral power at the very low frequency; LF = spectral power at the low frequency; HF = spectral power at the high frequency; LF/HF = low to high frequency index ratio; Hemo5 = 5 minutes hemorrhage; Hemo10 = 10 minutes hemorrhage; Hemo15 = 15 minutes hemorrhage; Shock30 = 30 min after hemorrhagic shock; Shock60 = 60 min after hemorrhagic shock; R0 = immediately after fluid resuscitation; R60 = 60 min after fluid resuscitation; R120 = 120 min after fluid resuscitation; R180 = 180 min after fluid resuscitation

* = P<0.05 different from Baseline

†<0,05 different from CH5 and CH10

§ <0,05 different from CH5, CH10 e CH15

A = P<0.05 group NS different from group S.

Hemorrhage induced a significant decrease in RR interval which was maintained lower than baseline even after fluid resuscitation. The HRV increased during hemorrhage but returned to baseline levels during shock maintainance. The RMSSD in the S group increased significantly at Hemorrhage5 and later at Hemorrhage10 in the NS group.

The values of normalized LF and HF components of HRV did not present significant alterations. The LF and autonomic balance, expressed as LF/HF, decreased during hemorrhage and increased after hemorrhage (Shock30) and resuscitation (R60, R120 and R180) in the S group, but not significantly.

### Hemodynamic changes

**Fig 3:**
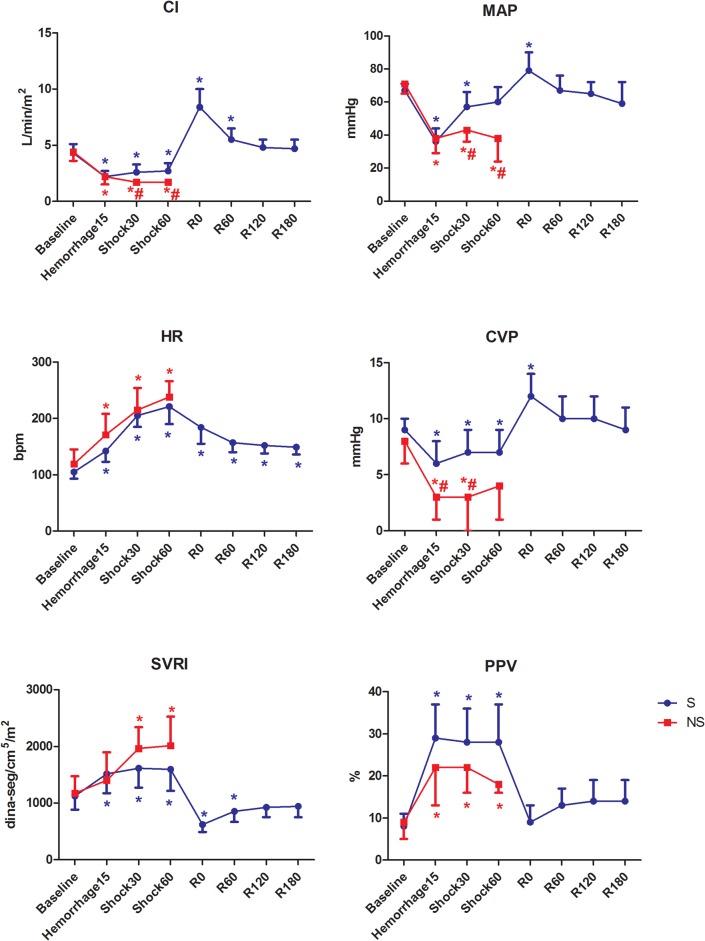
Hemodynamic variables in pigs submitted to acute hemorrhagic shock and fluid resuscitation. S = survivor group. NS = non-survivor group; * = P<0.05 different from baseline; # = p<0.05 NS group different from the S group (t test).

The HR increased significantly after hemorrhage in both groups (Fig 3). This parameter decreased significantly after fluid resuscitation compared with Shock60, but remained higher than baseline.

The MAP, CVP and CI decreased significantly after hemorrhage. The non-surviving animals presented significantly lower values of MAP and CI at Shock30 and Shock60, and CVP at Hemorrhage15 and Shock30. The SVRI increased significantly after hemorrhage and decreased after fluid resuscitation (R0 and R60).

The PPV increased significantly after hemorrhage and returned to baseline levels after fluid resuscitation (R0 to R180).

### Blood gases and electrolytes

**Fig 4:**
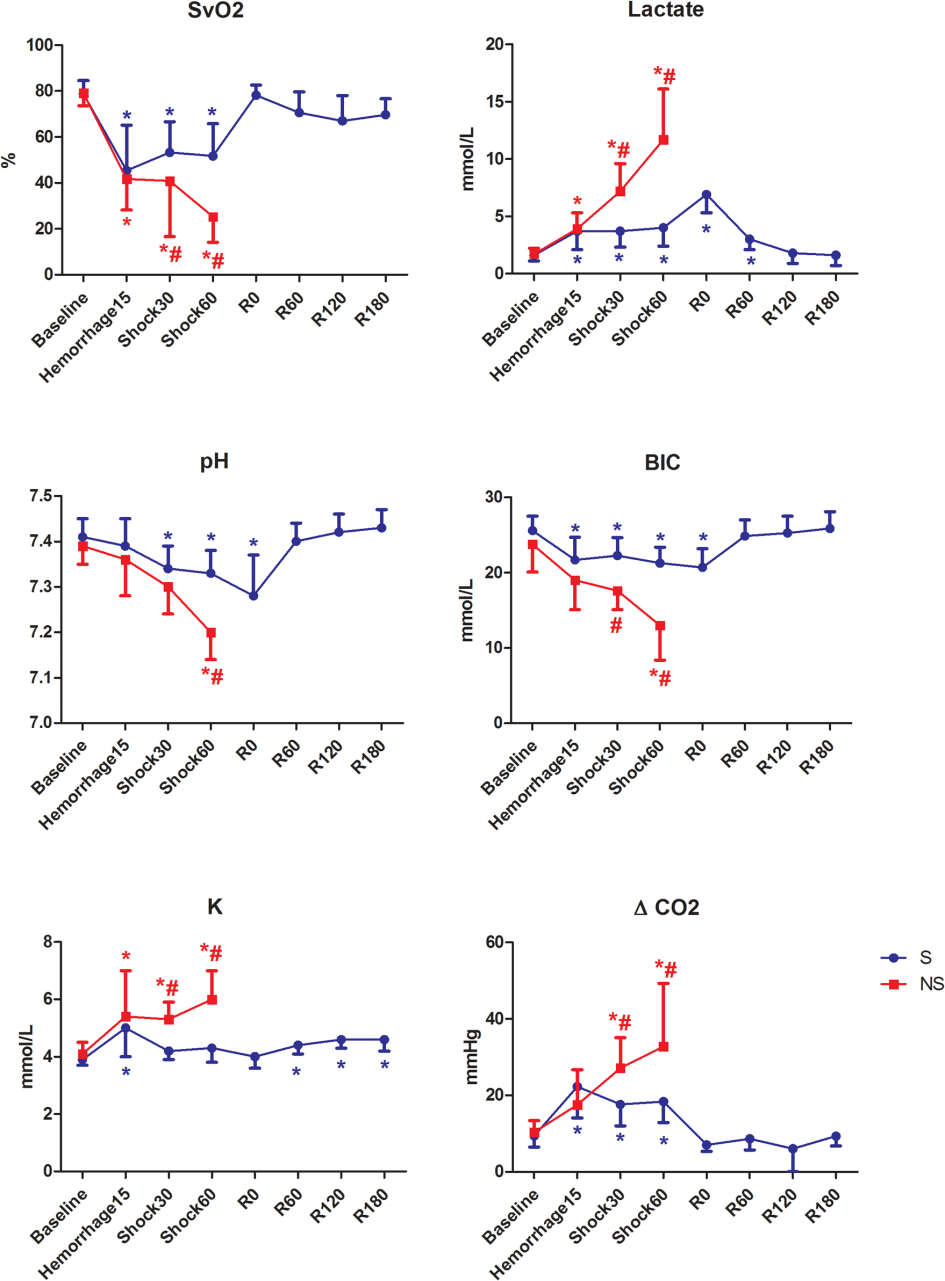
Metabolic variables in pigs submitted to acute hemorrhagic shock and fluid resuscitation. S = survivor group. NS = non-survivor group; * = P<0.05 different from baseline; # = p<0.05 NS group different from the S group (t test).

Hemorrhage induced a metabolic acidosis with a significant decrease in arterial pH and HCO_3_^-^ which was maintained until 60 min after fluid resuscitation. The NS animals presented lower values of pH at HS60 and lower values of HCO_3_^-^ at HS30 and HS60 compared with the S group (Fig 4).

The veno-arterial carbon dioxide tension difference increased significantly after hemorrhage, decreasing to baseline values after resuscitation. The NS group presented higher values compared with the S group at Shock30 and Shock60 (p = 0.0125 and 0.0127, respectively).

The SvO_2_ decreased significantly after hemorrhage and recovered baseline values after treatment. The NS group presented lower values at Shock60, compared with the S group.

Arterial lactate and potassium increased significantly after hemorrhage and the NS animals presented higher lactate potassium than the S animals.

When using the multivariable logistic regression analysis, neither HRV nor any hemodynamic or metabolic variable was selected as a predictor factor of mortality.

## Discussion

In this study, we evaluated heart rate variability metrics from EKG spectral analysis in pigs submitted to a severe hemorrhagic shock and fluid resuscitation. Time-domain indexes such as variance, SD of RRI and RMSSD were able to track volume status early, at 5 minutes of hemorrhage, returning to baseline values during shock and post-resuscitation periods. Non-survivor animals tended to increase these indexes later during hemorrhage. However, contrary to our hypothesis, HRV metrics were not able to distinguish survivor from non-survivors, while the hemodynamic and metabolic indices, such as CI and MAP, were lower, and serum lactate and veno-arterial carbon dioxide gradient were higher in non-survivors animals.

To our knowledge, this is the first study that compares HRV metrics to traditional vital signs between survivors and non-survivors in a severe model of controlled hemorrhage. Several studies have attempted to use different HRV metrics to detect hemorrhage or to predict injury severity in patients and animals. In trauma patients, some complexity domain measures as sample entropy and fractal dimension by curve length were able to distinguish patients receiving lifesaving interventions [18,19]. In a study with a small number of trauma patients, the LF and LF/HF ratio were higher and HF was lower in surviving patients [5]. However, a more recent study showed that only VLF predicted survival in traumatic brain injury patients [20]. In animal studies, it was observed loss of complexity and vagal withdrawal during hemorrhage [21–23].

The HRV has been proposed in both human and animal studies as a non-invasive surrogate for sympathetic/vagal activity [5,24,25]. Many studies have proposed that the LF provides an index of cardiac sympathetic tone, the HF reflects an index of parasympathetic tone, and the LF/HF ratio indicates the “sympatho-vagal balance”. However, recent findings showed that LF seems to provide not an index of cardiac sympathetic tone but of baroreflex function [26]. The HF and some time-domain components of HRV seems to decrease inversely in relation to the direct measurement of sympathetic nerve activity, while the LF does not change [21,27].

Early studies using direct neural measurements in conscious and anesthetized animals showed a two-phase physiological response to hemorrhage, an initial sympathoexcitation that maintains blood pressure, and a late sympathoinhibition with an increase in cardiac vagal drive causing a decrease in blood pressure [28]. More recent studies using lower body negative pressure (LBNP) in humans have shown that these increases in sympathetic activity occur at low levels of central volemia, before alterations in blood pressure or heart rate [25]. Later, different mechanisms were suggested to contribute to hemodynamic decompensation. Although some studies associated a sympathetic neural withdrawal with hemodynamic decompensation and hypotension [29], some recent data have demonstrated that it does not necessarily occur in all decompensating individuals [25]. Other factors, such as impairment of arterial baroreflex control over sympathetic vasomotor activity and changes in sympathetic nervous activation pattern, seem to contribute to the onset of the hemodynamic decompensation. It is important to note that in these studies with healthy humans submitted to LBNP, arterial pressure is compensated until syncope occurs. In our study, hemorrhage was performed at a rate of 3 mL/kg/min, by removing 60% of the volemia in 15 minutes, with great changes in CI and MAP.

Our data showed a decrease in normalized LF and LF/HF ratio early during hemorrhage, increasing only during maintenance of shock. However, neither absolute values nor normalized values of LF and HF were different between survivors and non-survivors. The hemodynamic and metabolic variables differed at 30 min of hemorrhagic shock, but neither evaluated parameters were able to discriminate non-survivors prior to cardiovascular collapse. Our study reinforces the previous findings from Hinojosa-Laborde and colleagues that showed that HRV metrics were not able to discriminate low tolerant individuals during central hypovolemia [30].

An unexpected result was that LF and HF did not return to baseline values after fluid resuscitation in surviving animals, contrary to the results described by Batchinsky et al [21]. One possible explanation could be the higher HR observed in our study, which was maintained above baseline values even after resuscitation. The magnitude of tachycardia could have influenced HRV metrics [27]. Acute anemia after fluid resuscitation (hematocrit decreased from 27 ± 2% at baseline to 14 ± 2%) could have contributed to the maintenance of tachycardia. Heart rate increases linearly in response to acute anemia, even during isovolemic conditions, as a compensatory mechanism to maintain oxygen delivery to the tissues. [31,32]. An additional group treated with blood instead of crystalloid solution could have resulted in different HRV responses. However, our objective was focused on the different HRV responses between survivors and non-survivors, and we did not aim to investigate the effects of different fluids on resuscitation.

One could question if fluid resuscitation was sufficient to restore normovolemia, as we opted to resuscitate with lactated Ringer´s solution with no reinfusion of shed blood. The crystalloid solution often restores normovolemia and rapidly leaks to the extravascular compartment. Our assumption was that the HRV metrics could detect hypovolemia after fluid extravasation. In fact, CI and MAP decreased slowly after resuscitation, while LF increased.

Hemodynamically, we observed a more pronounced decrease in MAP and CI after hemorrhage in non-survivors. After fluid resuscitation, both MAP and CI increased and remained near baseline values. The HR increased during hemorrhagic shock and decreased after treatment but persisted higher than baseline at all time-points, even immediately after fluid infusion. The elevated HR could be reflecting hypovolemia, despite filling pressures, such as PAWP and CVP, were near baseline values, and the PPV, a dynamic parameter of fluid responsiveness, had decreased after fluid resuscitation and increased only after 120 minutes. However, the volume of fluid employed to replace blood (3 X shed blood volume) could be insufficient, considering that a recent study demonstrated an intravascular retention of Ringer´s lactate below 20% [33].

Metabolic alterations observed after hemorrhagic shock were restored to normal values 60 min after fluid resuscitation. Non-survivor animals presented a lower arterial pH, HCO3-, BE and SvO_2_, as well as higher serum lactate and potassium than survivors. Similarly, Torres et al observed a worst metabolic acidosis and a higher level of potassium in non-survivors rats during prolonged hemorrhage, although HCO3-, BE and lactate were similar between survivors and non-survivors [34]. Serum potassium has been shown to increase early during hemorrhagic shock and its increase is related to mortality in animal models of hemorrhagic shock. A high correlation between potassium and lactate, SvO_2_, pH and ΔCO_2_ was also observed [35,36].

Early recognition of physiological deterioration is an ongoing challenge in critical care medicine. In our study, none of the studied HRV parameters was able to accurately identify hemodynamically unstable animals before cardiovascular collapse and death. Despite some promising results with HRV as a tool to identify hypovolemia or predict mortality [5,37], its usefulness remains controversial. Between and within subjects, variability and technical difficulties related to EKG recording and HRV calculation are the principal limitations. High inter-subject and low reproducibility were observed in time and frequency domain metrics in resting healthy volunteers, hampering the establishment of a “normal” range for these variables [38]. The EKG signals must be free of ectopy and have low level of noise. The EKG signal should also be stationary, which is very difficult to achieve during hemodynamic instability [8]. At this moment, HRV calculations are made off-line, and a real-time system is required to be clinically valuable.

The major limitation of this study is the small number of non-surviving animals. If the observation period of the hemorrhagic shock was longer than 60 minutes, the differences in cardiovascular function and HRV would be more evident between groups (with a possible increase in the number of deaths), but a period of shock greater than one hour would be incompatible with the clinical setting. In addition, although power calculations were <20% for the HRV variables, the hemodynamic and metabolic data analysis showed a power >80%. However, a sample size for HRV metrics to achieve 80% power results in a total number of animals greater than 200, which is impracticable for ethical reasons in an experimental study. Anesthesia could be another limitation as HRV power spectra in all components were shown to be reduced by isoflurane anesthesia [39]. Lung ventilation is also another limiting factor, as the HF component is highly dependent of respiratory frequency. However, the effect of anesthetics was constant all over the study, since isoflurane concentration was not changed throughout the entire experiment, as well as the ventilator settings (tidal volume and respiratory frequency). Any anesthetic protocol, regardless if inhalant or intravenous anesthesia, could have had some effect on the HRV power spectra. According to Kato et al. [39], the dose-dependent decrease in the HRV caused by isoflurane is associated with a MAC of 2, and in this study, we used a MAC of 0.7 for pigs [40], which we assumed to have a minor influence. Finally, this is an experimental study performed in young healthy female pigs with no additional co-morbidities. Several studies have demonstrated age and gender related differences in the HRV metrics and cardiovascular responses to different physiological conditions [41,42].

In conclusion, in our study, HRV metrics were not able to discriminate survivors from non-survivors. Moreover, metabolic variables along with CI and MAP were more reliable to reflect hemorrhagic shock severity than HRV metrics. To date, hemorrhagic shock severity cannot be accurately assessed by one specific parameter or index, and a combined assessment of hemodynamic, metabolic and EKG derived parameters is required.

## Supporting Information

S1 Dataset (PDF)
