# Supplementary material for: Heart Rate Variability Analysis in an Experimental Model of Hemorrhagic Shock and Resuscitation in Pigs
Source: PLoS One. 2015 Aug 6;10(8):e0134387. doi: 10.1371/journal.pone.0134387 (PMC4527725; doi:10.1371/journal.pone.0134387)
Supplement: S1 Dataset — (PDF) [file pone.0134387.s001.pdf]

VLFabs

	S-Basal	S-Hemo5	S-Hemo10	S-Hemo15	S-Hemo20	S-Shock30	S-Shock60	S-R 0	S-R 60	S-R 120	S-R 180	NS-Basal	NS-Hemo5	NS-Hemo10	NS-Hemo15	NS-Hemo20	NS-Shock30	NS-Shock60
	3,24	1,53	0,31	1,8	3,08	0,43	0,44	0,15	0,75	0,67	1,01	3,4	3,66	2,31	69,96	34,04	7,32	0,81
										4,25	2,13	0,18	18,98	93,61	3105,84		1,76	0,02
	2,23	8,97	0,76	3,73	0,74	0,42	0,9	0,36	3,6			0,44	2,68	24,04	0,46	0,29	0,16	1,26
										2,08	2,32	10,22	8,31	13,92	0,48	1,62	1,09	0,03
	4,2	4,02	1,6	1,15	1,7	0,3	0,14	0,38	1,44	2,31	2,33	0,46	1,68	3,57	21,56	27,11	0,15	
	10,73	9,9	3,58	3,27	13,43	4,99	3,93	1,13	2,46	1,34	0,65	0,48	3,66	9,81	0,43	1,1	0,12	
	0,17	0,26	0,14	0,76		0,34	0,32	1,04	1,23	0,7	0,73	1,63	3,19	11,88	5,28	2,04	0,21	
	0,86	1,51	1,39	8,29	1,65	1,24	0,36	0,32	0,44									
	0,8	24,67	0,8	3,58	3,83	0,73	1,53	1,26	2,36	8,33	7,68							
	0,09	0,54	0,12	0,11	0,1	0,13	0,03	0,13	0,1	0,23	0,28							
	1,41	25,23	0,49	0,33	0,29	0,18	0,12	0,08	0,39	0,44	0,51							
	7,35	7,92	2,09	4,23		0,64		0,31	0,52	0,98	1,39							
							0,47	0,62	1,6	2,54	4,01							
**Mean**	**3,11**	**8,46**	**1,13**	**2,73**	**3,10**	**0,94**	**0,82**	**0,53**	**1,35**	**2,17**	**2,09**	**2,40**	**6,02**	**22,73**	**457,72**	**11,03**	**1,54**	**0,53**
**SD**	**3,48**	**9,38**	**1,08**	**2,47**	**4,37**	**1,46**	**1,18**	**0,43**	**1,08**	**2,36**	**2,15**	**3,63**	**6,09**	**32,07**	**1167,98**	**15,31**	**2,62**	**0,61**

LFabs

	S-Basal	S-Hemo5	S-Hemo10	S-Hemo15	S-Hemo20	S-Shock30	S-Shock60	S-R 0	S-R 60	S-R 120	S-R 180	NS-Basal	NS-Hemo5	NS-Hemo10	NS-Hemo15	NS-Hemo20	NS-Shock30	NS-Shock60
	5,35	1,17	14	0,64	1,38	0,77	0,82	0,25	1,34	1,41	1,98	9,21	7,5	4,46	123,97	49,42	15,98	1,14
										5,78	3,34	0,33	48,05	210,81	5783,43		3,83	0,04
	4,6	22,38	1	2,91	0,37	0,68	1,34	0,64	4,69			0,92	9,8	71,66	0,59	0,19	0,29	4,05
										5,46	6,22	21,18	24,08	59,88	1,12	0,71	1,62	0,05
	8,28	2,19	13	0,39	1,17	0,48	0,21	0,84	3,23	5,46	5,72	1,24	1,55	6,29	52,57	75,11	0,56	
	19,32	7,83	39	7,7	22,61	13,08	11,24	3,21	5,06	2,16	1,05	1,04	3,28	4,85	0,39	2,73	0,22	
	0,43	0,25	5	0,83		0,59	0,59	1,55	1,93	1,28	1,34	2,53	1,57	21,8	1,57	0,54	0,27	
	1,94	1,32	41	4,74	2,78	2,13	0,63	0,47	0,79									
	0,59	23,26	16	2,64	5,16	0,17	2,67	0,54	1,64	4,7	7,92							
	0,13	0,11	20	0,13	0,08	0,09	0,04	0,08	0,08	0,11	0,19							
	1,28	62,82	23	0,36	0,15	0,07	0,07	0,06	0,7	0,55	0,7							
	8,66	3,07	46	1,98		0,6		0,33	0,46	0,79	1,1							
**Mean**	**5,06**	**12,44**	**21,80**	**2,23**	**4,21**	**1,87**	**1,96**	**0,80**	**1,99**	**2,77**	**2,96**	**5,21**	**13,69**	**54,25**	**851,95**	**21,45**	**3,25**	**1,32**
**SD**	**5,93**	**19,77**	**15,43**	**2,42**	**7,63**	**3,98**	**3,58**	**0,95**	**1,76**	**2,30**	**2,72**	**7,68**	**17,05**	**74,29**	**2175,07**	**32,65**	**5,76**	**1,89**

HFabs

	S-Basal	S-Hemo5	S-Hemo10	S-Hemo15	S-Hemo20	S-Shock30	S-Shock60	S-R 0	S-R 60	S-R 120	S-R 180	NS-Basal	NS-Hemo5	NS-Hemo10	NS-Hemo15	NS-Hemo20	NS-Shock30	NS-Shock60
	0,71	1,12	0,9	0,29	0,46	0,37	0,45	0,56	0,61	0,51	0,59	3,15	3,45	2,01	730,41	428,32	2,88	0,42
										0,69	0,74	1,28	98,29	1954,13	21531,12		2,34	0,47
	15,47	39	140,73	176,12	0,49	0,5	0,66	0,74	0,67			2,6	87,08	634,81	19,52	0,26	0,26	2,09
										2	2,08	9,81	85,01	354,97	12,49	1,24	0,94	0,16
	14,08	7,71	8,21	2,15	14,46	0,44	0,36	0,63	1,24	1,49	1,42	1,05	1,94	3,91	869,52	1886,39	0,74	
	3,29	1,87	1,67	3,7	39,18	3,76	2,93	2,31	1,84	1,68	1,29	1,85	1,83	17,86	0,44	3,15	0,58	
	1,19	1,39	1,98	51,27		0,42	0,42	0,89	1,79	0,34	0,4	1,22	0,53	1392,54	0,7	0,44	0,25	
	0,79	2,56	0,48	17,62	0,27	0,35	0,42	0,33	0,33									
	3,38	3,7	2,51	40,67	2,12	0,69	1,59	0,38	0,91	1,22	2,16							
	0,6	0,88	0,32	0,26	0,24	0,24	0,22	0,42	0,5	0,38	0,35							
	7,86	461,35	34,02	0,52	2,77	0,32	0,32	0,31	1,61	1,06	1,23							
	12,13	7,13	0,39	272,81		0,29		0,41	0,6	1,34	1,53							
							4,62	1,03	1,03	1,83	2,8							
**Mean**	**5,95**	**52,67**	**19,12**	**56,54**	**7,50**	**0,74**	**1,20**	**0,73**	**1,01**	**1,14**	**1,33**	**2,99**	**39,73**	**622,89**	**3309,17**	**386,63**	**1,14**	**0,79**
**SD**	**5,94**	**144,05**	**43,95**	**93,15**	**13,66**	**1,07**	**1,47**	**0,58**	**0,54**	**0,59**	**0,79**	**3,10**	**47,33**	**771,49**	**8043,89**	**754,32**	**1,04**	**0,88**

VLF%

	S-Basal	S-Hemo5	S-Hemo10	S-Hemo15	S-Hemo20	S-Shock30	S-Shock60	S-R 0	S-R 60	S-R 120	S-R 180	NS-Basal	NS-Hemo5	NS-Hemo10	NS-Hemo15	NS-Hemo20	NS-Shock30	NS-Shock60
	28	33	14	50	38	23	21	11	22	21	23	19	22	24	21	41	25	22
										32	28	8	14	9	12		20	6
	6	4	1	11	28	21	23	17	28			9	16	14	13	30	17	16
										18	19	18	8	9	8	39	23	9
	14	27	13	29	30	19	13	18	18	22	21	15	29	20	6	19	9	
	27	48	39	20	21	21	19	15	21	21	17	13	39	18	26	23	14	
	8	11	5	21		21	20	23	19	25	24	24	47	8	63	43	17	
	20	28	41	45	29	27	20	20	22									
	16	40	16	15	30	27	22	47	39	43	40							
	11	24	20	22	25	26	12	19	13	27	27							
	12	10	23	27	32	24	21	15	20	20	19							
	21	24	46	20		32		26	29	28	30							
							7	18	26	24	26							
**Mean**	**16,30**	**24,90**	**21,80**	**26,00**	**29,13**	**24,10**	**17,80**	**20,82**	**23,36**	**25,55**	**24,91**	**15,14**	**25,00**	**14,57**	**21,29**	**32,50**	**17,86**	**13,25**
**SD**	**7,56**	**13,72**	**15,43**	**12,50**	**4,97**	**3,93**	**5,27**	**9,57**	**6,93**	**7,06**	**6,49**	**5,70**	**14,12**	**6,27**	**19,70**	**10,03**	**5,43**	**7,18**

LF%

	S-Basal	S-Hemo5	S-Hemo10	S-Hemo15	S-Hemo20	S-Shock30	S-Shock60	S-R 0	S-R 60	S-R 120	S-R 180	NS-Basal	NS-Hemo5	NS-Hemo10	NS-Hemo15	NS-Hemo20	NS-Shock30	NS-Shock60
	57	34	27	28	36	45	45	20	47	51	53	57	51	50	37	22	56	40
										52	50	17	28	11	18		49	11
	12	10	2	12	23	36	36	34	44			22	12	6	14	24	37	53
										56	55	45	29	9	12	19	37	18
	28	14	9	10	22	35	23	45	48	55	59	40	30	43	14	20	27	
	48	40	39	51	45	55	60	40	48	37	30	30	32	15	35	32	27	
	22	13	10	18		40	40	40	34	53	52	44	26	4	24	22	25	
	48	30	35	35	58	55	37	35	46									
	11	30	27	20	46	9	39	26	30	39	45							
	16	8	21	21	20	18	15	12	11	14	22							
	11	8	12	28	14	14	14	12	32	27	30							
	30	13	34	5		35		37	28	24	26							
							19	35	51	54	52							

You created this PDF from an application that is not licensed to print to novaPDF printer (http://www.novapdf.com)

	S-Basal	S-Hemo5	S-Hemo10	S-Hemo15	S-Hemo20	S-Shock30	S-Shock60	S-R 0	S-R 60	S-R 120	S-R 180	NS-Basal	NS-Hemo5	NS-Hemo10	NS-Hemo15	NS-Hemo20	NS-Shock30	NS-Shock60
**Mean**	**28,30**	**20,00**	**21,60**	**22,80**	**33,00**	**34,20**	**32,80**	**30,55**	**38,09**	**42,00**	**43,09**	**36,43**	**29,71**	**19,71**	**22,00**	**23,17**	**36,86**	**30,50**
**SD**	**17,19**	**12,10**	**12,76**	**13,46**	**15,57**	**16,07**	**14,77**	**11,40**	**12,23**	**14,76**	**13,35**	**14,11**	**11,47**	**18,74**	**10,34**	**4,67**	**11,89**	**19,43**

HF%

	S-Basal	S-Hemo5	S-Hemo10	S-Hemo15	S-Hemo20	S-Shock30	S-Shock60	S-R 0	S-R 60	S-R 120	S-R 180	NS-Basal	NS-Hemo5	NS-Hemo10	NS-Hemo15	NS-Hemo20	NS-Shock30	NS-Shock60
	14	32	59	21	25	33	34	69	32	28	25	24	27	26	42	37	19	38
										16	22	75	58	81	70		30	84
	82	85	97	77	49	43	41	50	28			69	72	80	73	45	46	31
										26	26	37	63	82	80	41	40	73
	58	59	78	60	48	46	64	38	35	23	21	45	41	37	80	61	63	
	25	12	21	29	33	24	21	45	31	43	53	58	30	66	39	45	59	
	69	76	85	62		39	40	37	47	22	24	32	26	88	13	35	58	
	32	42	23	20	12	18	42	44	31									
	73	30	57	65	25	64	39	27	31	18	16							
	74	69	59	57	56	56	74	69	76	59	51							
	77	82	65	45	54	63	66	73	48	53	51							
	49	62	20	76		33		37	43	48	44							
							74	47	23	22	22							
**Mean**	**55,30**	**54,90**	**56,40**	**51,20**	**37,75**	**41,90**	**49,50**	**48,73**	**38,64**	**32,55**	**32,27**	**48,57**	**45,29**	**65,71**	**56,71**	**44,00**	**45,00**	**56,50**
**SD**	**24,15**	**24,73**	**27,31**	**21,38**	**16,21**	**15,67**	**18,45**	**15,21**	**14,71**	**15,26**	**14,26**	**19,28**	**18,92**	**24,50**	**25,71**	**9,27**	**16,41**	**25,96**

LF(nu)

	S-Basal	S-Hemo5	S-Hemo10	S-Hemo15	S-Hemo20	S-Shock30	S-Shock60	S-R 0	S-R 60	S-R 120	S-R 180	NS-Basal	NS-Hemo5	NS-Hemo10	NS-Hemo15	NS-Hemo20	NS-Shock30	NS-Shock60
	82	50	33	60	64	60	59	24	61	66	70	71	67	67	47	51	76	54
										78	72	19	33	14	21		64	12
	14	12	2	15	34	48	52	42	66			25	16	9	18	37	46	64
										70	70	57	32	10	14	33	52	21
	34	21	11	16	36	46	28	55	60	72	75	48	44	55	17	30	32	
	71	77	61	65	60	71	76	49	63	48	38	35	54	21	47	41	33	
	25	15	11	26		53	52	55	44	72	70	61	59	7	67	42	35	
	62	48	63	70	83	77	51	47	62									
	13	53	33	28	65	17	52	54	52	72	75							
	19	11	27	26	26	25	17	16	13	20	32							
	13	9	20	42	25	21	19	15	41	35	37							
	39	23	67	15		56		52	42	36	39							
							21	44	70	72	71							
**Mean**	**37,20**	**31,90**	**32,80**	**36,30**	**49,13**	**47,40**	**42,70**	**41,18**	**52,18**	**58,27**	**59,00**	**45,14**	**43,57**	**26,14**	**33,00**	**39,00**	**48,29**	**37,75**
**SD**	**25,75**	**23,35**	**23,51**	**21,48**	**21,57**	**20,61**	**20,03**	**15,43**	**16,41**	**19,87**	**18,01**	**19,41**	**17,80**	**24,48**	**20,55**	**7,46**	**16,88**	**25,14**

HF(nu)

	S-Basal	S-Hemo5	S-Hemo10	S-Hemo15	S-Hemo20	S-Shock30	S-Shock60	S-R 0	S-R 60	S-R 120	S-R 180	NS-Basal	NS-Hemo5	NS-Hemo10	NS-Hemo15	NS-Hemo20	NS-Shock30	NS-Shock60
	18	50	67	40	36	40	41	76	39	34	30	29	33	33	53	49	24	46
										22	28	81	67	86	79		36	88
	86	88	98	85	66	52	48	58	34			75	84	91	82	63	54	36
										30	30	43	68	90	86	67	48	79
	66	79	89	84	64	54	72	45	40	28	25	52	56	45	83	70	68	
	29	23	39	35	40	29	24	51	37	52	62	65	46	79	53	59	67	
	75	85	89	74		47	48	45	56	28	30	39	41	93	33	58	65	
	38	52	37	30	17	23	49	53	38									
	87	47	67	72	35	83	48	46	48	28	25							
	81	89	73	74	74	75	83	84	87	80	68							
	87	91	80	58	75	79	81	85	59	65	63							
	61	77	33	85		44		48	58	64	61							
							79	56	30	28	29							
**Mean**	**62,80**	**68,10**	**67,20**	**63,70**	**50,88**	**52,60**	**57,30**	**58,82**	**47,82**	**41,73**	**41,00**	**54,86**	**56,43**	**73,86**	**67,00**	**61,00**	**51,71**	**62,25**
**SD**	**25,75**	**23,35**	**23,51**	**21,48**	**21,57**	**20,61**	**20,03**	**15,43**	**16,41**	**19,87**	**18,01**	**19,41**	**17,80**	**24,48**	**20,55**	**7,46**	**16,88**	**25,14**

LF/HF

	S-Basal	S-Hemo5	S-Hemo10	S-Hemo15	S-Hemo20	S-Shock30	S-Shock60	S-R 0	S-R 60	S-R 120	S-R 180	NS-Basal	NS-Hemo5	NS-Hemo10	NS-Hemo15	NS-Hemo20	NS-Shock30	NS-Shock60
	10,18	1,31	0,93	2,8	2,91	2,42	2,33	0,5	2,29	3,01	4	3,72	2,84	3,02	2,04	3,9	6,58	3,97
										8,94	4,72	0,26	0,51	0,24	0,37		3,51	0,16
	0,27	0,22	0,02	0,21	0,66	1,37	2,03	0,92	6,83			0,39	0,21	0,11	0,28	0,76	1,28	3,02
										3,39	3,58	2,5	0,58	0,13	0,19	0,6	2,01	0,34
	0,64	0,3	0,13	0,2	0,79	1,32	0,62	1,66	3,15	4,14	4,9	1,16	0,94	1,69	0,77	0,9	0,82	
	13,6	6,95	3,14	2,65	2,2	4,6	5,91	2,27	3,04	1,38	0,85	0,67	2,02	0,5	1,07	0,85	0,72	
	0,4	0,21	0,12	0,62		1,69	1,59	2,12	1,06	4,28	3,66	3,47	3,09	0,11	2,87	1,54	1,13	
	2,82	1,87	2,6	6,99	11,5	7,42	1,92	1,56	2,68									
	0,17	5,03	0,58	0,94	2,6	0,26	1,8	1,6	1,78	4,54	5,48							
	0,26	0,13	0,42	0,46	0,44	0,42	0,22	0,21	0,16	0,28	0,56							
	0,17	0,1	0,33	1,15	0,39	0,31	0,27	0,21	0,87	0,65	0,66							
	0,76	0,41	2,68	0,3		2,3		1,37	0,84	0,61	0,72							
							0,26	1,33	3,33	3,77	3,5							
**Mean**	**2,93**	**1,65**	**1,10**	**1,63**	**2,69**	**2,21**	**1,70**	**1,25**	**2,37**	**3,18**	**2,97**	**1,74**	**1,46**	**0,83**	**1,08**	**1,43**	**2,29**	**1,87**
**SD**	**4,86**	**2,40**	**1,22**	**2,11**	**3,70**	**2,24**	**1,69**	**0,71**	**1,83**	**2,49**	**1,90**	**1,47**	**1,18**	**1,12**	**1,01**	**1,25**	**2,12**	**1,91**

Mean

	S-Basal	S-Hemo5	S-Hemo10	S-Hemo15	S-Hemo20	S-Shock30	S-Shock60	S-R 0	S-R 60	S-R 120	S-R 180	NS-Basal	NS-Hemo5	NS-Hemo10	NS-Hemo15	NS-Hemo20	NS-Shock30	NS-Shock60
	676,85	668,31	617,35	545,05	491,43	275,57	266,73	260,56	336,72	356,6	372,07	353,15	297,48	266,99	259,59	263,42	271,71	290,43
										377,07	371,04	443,5	453,25	419,82	301,14		336,34	255,58
	505,96	485,3	471,38	378,66	284,43	264,68	255,81	324,85	369,88			586,5	528,87	471,75	372,52	272,39	236,78	252,07
										361,24	367,03	502,88	490,9	476,56	448,31	401,93	361,97	218,53
	613,37	579,78	502,07	416,43	331,06	278,73	260,98	304,36	350,2	431,49	421,49	528,15	534,02	513,62	447,87	257,21	250,56	
	466,18	470,01	430,37	403,6	321,66	270,11	246,98	289,53	387,71	468,75	461,58	654,11	608,65	490,3	401,66	349,9	296,6	
	584,08	585,44	535,43	437,6		385,92	379,5	461,45	488,27	401,15	407,69	585,79	468,08	427,88	314,59	248,81	242,11	
	670,17	642,03	627,13	451,41	330,5	289,22	265,37	326,17	380,35									
	542,28	604,1	524,84	524,84	319,74	306,52	265,38	324,4	348,82	384,92	429,33							
	508,13	484,91	424,49	348,98	291,84	287,9	258,12	313,28	369,18	355,38	360,69							

You created this PDF from an application that is not licensed to print to novaPDF printer (http://www.novapdf.com)

	S-Basal	S-Hemo5	S-Hemo10	S-Hemo15	S-Hemo20	S-Shock30	S-Shock60	S-R 0	S-R 60	S-R 120	S-R 180	NS-Basal	NS-Hemo5	NS-Hemo10	NS-Hemo15	NS-Hemo20	NS-Shock30	NS-Shock60
	601,89	539,17	496,75	396,34	347,19	279,52	278,77	291,46	405,25	442,12	459,18							
	608,67	547,15	390,69	398,64		285,78		355,35	389,85	424,14	434,31							
							277,42	319,15	375,05	390,83	407,61							
**Mean**	**577,76**	**560,62**	**502,05**	**430,16**	**339,73**	**292,40**	**275,51**	**324,60**	**381,93**	**399,43**	**408,37**	**522,01**	**483,04**	**438,13**	**363,67**	**298,94**	**285,15**	**254,15**
**SD**	**70,65**	**67,79**	**78,24**	**62,31**	**64,70**	**34,83**	**37,75**	**51,65**	**40,56**	**37,80**	**36,59**	**100,63**	**96,68**	**82,42**	**74,08**	**62,33**	**48,69**	**29,39**

SD

	S-Basal	S-Hemo5	S-Hemo10	S-Hemo15	S-Hemo20	S-Shock30	S-Shock60	S-R 0	S-R 60	S-R 120	S-R 180	NS-Basal	NS-Hemo5	NS-Hemo10	NS-Hemo15	NS-Hemo20	NS-Shock30	NS-Shock60
	7,35	5,91	24,63	27,3	25,79	15,69	5,74	5,71	6,6	5,23	9,19	4,11	23,51	22,97	6,87	19,07	18,73	37,58
										11,14	12,12	11,71	15,01	28,71	34,88		59,11	7,19
	10,85	15,05	18,09	53,46	10,57	3,12	2,93	9,35	9,42			5,2	26,29	34,42	34,82	12,71	19,04	6,77
										4,25	16,53	14,97	12,56	26,41	11,51	5,49	40,99	4,17
	12,13	46,15	25,3	23,94	40,48	13,38	6,88	16,46	30,03	8,03	8,65	4,85	11,57	21,47	44,32	38,52	7,04	
	19,83	23,86	25,19	4,68	40,73	10,02	6,41	42,27	22,68	15,67	5,76	8,19	17,89	57,05	8,44	18,92	17,26	
	10,7	19,86	20,08	37,34		18,26	30,79	9,35	6,47	7,97	4,8	16,25	57,9	33,02	38,04	5,39	22,09	
	8,94	47,09	35,12	34,3	15,87	18,59	6,37	21,83	10,98									
	31,17	12,19	45,74	45,74	51,53	18,78	9,97	9,93	18,12	20,73	20,81							
	7,46	24,59	21,49	24,87	11,61	16,76	8,39	7,35	8,56	9,41	8,04							
	16,75	60,88	27,05	25,19	8,51	11,02	11,88	17,03	17,6	6,82	4,05							
	7,48	30,84	36,06	35,96		6,86		4,38	9,4	3,31	4,41							
							7,23	5,26	6,11	6,49	5,52							
**Mean**	**13,27**	**28,64**	**27,88**	**31,28**	**25,64**	**13,25**	**9,66**	**13,54**	**13,27**	**9,00**	**9,08**	**9,33**	**23,53**	**32,01**	**25,55**	**16,68**	**26,32**	**13,93**
**SD**	**7,53**	**17,57**	**8,57**	**13,43**	**16,61**	**5,39**	**7,81**	**11,02**	**7,82**	**5,19**	**5,41**	**5,02**	**16,11**	**12,03**	**15,92**	**12,30**	**17,67**	**15,82**

Variance

	S-Basal	S-Hemo5	S-Hemo10	S-Hemo15	S-Hemo20	S-Shock30	S-Shock60	S-R 0	S-R 60	S-R 120	S-R 180	NS-Basal	NS-Hemo5	NS-Hemo10	NS-Hemo15	NS-Hemo20	NS-Shock30	NS-Shock60
	53,99	34,96	606,65	745,07	664,91	246,22	32,97	32,56	43,61	27,33	84,48	16,86	552,69	527,82	47,18	363,56	350,65	1412,09
										124,11	146,98	137,12	225,41	824,45	1216,59		3494,28	51,64
	117,78	226,45	327,4	2857,96	111,63	9,73	8,59	87,33	88,73			27	691,34	1184,7	1212,74	161,55	362,43	45,78
										18,04	273,2	224,24	157,73	697,61	132,42	30,09	1680,27	17,41
	147,2	2129,4	640,24	572,97	1638,43	179,01	47,3	270,8	901,59	64,48	74,86	23,53	133,87	460,97	1964,49	1483,95	49,55	
	393,06	569,07	634,77	21,9	1658,98	100,42	41,08	1786,9	514,34	245,66	33,16	67,08	319,97	3255,04	71,2	357,85	298,05	
	114,59	394,22	403,02	1394,14		333,31	948,2	87,38	41,8	63,46	23,06	264,2	3352,15	1090,5	1447,07	29,05	487,87	
	79,85	2217,43	1233,65	1176,58	251,95	345,67	40,52	476,75	120,53									
	971,67	148,59	2092,34	2092,34	2655,17	352,68	99,31	98,55	328,19	429,88	433,04							
	55,59	604,75	462,03	618,35	134,78	280,97	70,44	53,95	73,23	88,6	64,63							
	280,43	3706,55	731,87	634,71	72,43	121,47	141,24	290,08	309,89	46,48	16,4							
	55,99	951,1	1300	1293,08		47,01		19,19	88,39	10,95	19,44							
							52,33	27,69	37,35	42,09	30,45							
**Mean**	**227,02**	**1098,25**	**843,20**	**1140,71**	**898,54**	**201,65**	**148,20**	**293,74**	**231,60**	**105,55**	**109,06**	**108,58**	**776,17**	**1148,73**	**870,24**	**404,34**	**960,44**	**381,73**
**SD**	**284,04**	**1199,65**	**545,64**	**830,18**	**968,73**	**127,85**	**283,57**	**515,63**	**270,50**	**126,16**	**131,55**	**102,01**	**1154,65**	**966,82**	**777,57**	**549,41**	**1235,57**	**687,07**

RMSSD

	S-Basal	S-Hemo5	S-Hemo10	S-Hemo15	S-Hemo20	S-Shock30	S-Shock60	S-R 0	S-R 60	S-R 120	S-R 180	NS-Basal	NS-Hemo5	NS-Hemo10	NS-Hemo15	NS-Hemo20	NS-Shock30	NS-Shock60
	1,35	1,66	1,46	22,96	0,98	1,36	1,05	1,84	0,96	0,97	1,15	1,36	1,27	31,36	1,64	23,3	1,24	1,3
										1,19	1,28	2,11	8,97	23,31	9,17		3,69	1,99
	6,64	11,96	23,82	40,57	12,06	1,02	1,14	1,7	1,18			2,69	26,5	33,22	10,89	0,76	1,02	1,84
										1,47	2,28	3,6	14,68	30,32	4,83	1,41	1,24	0,95
	5,36	69,58	3,88	29,55	20,42	1,15	1,02	2,48	2,79	1,32	1,25	1,4	13,79	15,39	44,17	52,37	2,22	
	1,94	30,05	32,03	1,47	15,24	1,79	1,14	2,25	1,6	1,75	1,56	3,02	1,99	25,75	8,05	1,54	1,27	
	1,67	1,74	2,26	11,52		1,24	1,35	1,43	1,85	0,88	0,95	1,28	42,94	19,07	13,85	0,89	0,86	
	4	46,33	19,87	6,15	0,91	1,01	1,04	1,79	0,93									
	11,02	2,96	12,38	12,38	1,26	9,64	9,57	1,47	5,76	1,25	1,63							
	10,87	26,79	10,54	0,78	0,86	0,9	10,46	3,85	3,36	0,95	2,47							
	3,56	78,61	25,42	8,56	2,64	2,05	5,04	18,4	7,26	2,44	5,08							
	4,52	29,12	7,11	35,27		0,92		1,01	1,06	1,36	1,42							
							1,44	1,2	1,59	1,71	1,23							
**Mean**	**5,09**	**29,88**	**13,88**	**16,92**	**6,80**	**2,11**	**3,33**	**3,40**	**2,58**	**1,39**	**1,85**	**2,21**	**15,73**	**25,49**	**13,23**	**13,38**	**1,65**	**1,52**
**SD**	**3,50**	**27,62**	**10,78**	**14,25**	**7,89**	**2,67**	**3,74**	**5,03**	**2,12**	**0,45**	**1,17**	**0,92**	**14,75**	**6,65**	**14,21**	**21,06**	**1,00**	**0,48**

CI

	S-Basal	S-Hemo15	S-Shock30	S-Shock60	S-R 0	S-R 60	S-R 120	S-R 180	NS-Basal	NS-Hemo15	NS-Shock30	NS-Shock60
	3,5	1,9	2,6	2,6	7,3	4,5	3,9	3,6	4	1,6	1,5	
	5	2	3	2,9	10,9	5,7	5,3	5,5	4,6			
	4	1,9	2,3	2	7,8	5,6	4,5	4,6	2,9	1,3	1,5	1,6
	3,9	2,4	2,3	3,2	11,8	5,7	5,4	5	4,6	2,5	1,5	
	3,6	1,8	2	1,8	6,9	4,3	4	4,1	5,5	3	1,9	1,6
	3,4	1,9	2,2	2,1	8,3	6,8	6,1	4,7	5	2,9	1,9	1,9
	4,7	2,5	3	3,4	7,8	6	4,9	4,8	4,4	2,1		
	4,9	1,8	2,8	3,1	7,3	4,8	4,9	4,9				
	5,9	3,3	4,2	4,2	9,9	6,9	5,1	5,9				
	4,5	2,2	2,2	2,5	7,5	5,2	4,7	4,6				
	5,3	2,5	3,2	3,4	9	7,1	5,6	5,4				
	3,9	1,5	1,6	1,9	6,7	3,9	3,4	3,2				
	3,9	2,6	2,9	2,4	7,4	5,4	4,9	5,2				
**Mean**	**4,35**	**2,18**	**2,64**	**2,73**	**8,35**	**5,53**	**4,82**	**4,73**	**4,43**	**2,23**	**1,66**	**1,70**
**SD**	**0,77**	**0,47**	**0,66**	**0,71**	**1,59**	**1,01**	**0,74**	**0,75**	**0,82**	**0,69**	**0,22**	**0,17**

MAP

S-Basal	S-Hemo15	S-Shock30	S-Shock60	S-R 0	S-R 60	S-R 120	S-R 180	NS-Basal	NS-Hemo15	NS-Shock30	NS-Shock60
73	38	56	63	95	77	75	71	80	38	41	20
69	28	55	53	77	71	60	56	77	40		
61	43	60	70	61	59	59	47	71	28	55	53
72	39	63	63	83	72	69	61	64	50	36	
68	38	59	51	72	58	66	66	64	41	42	42
72	36	60	60	87	73	71	73	70	42	39	38

You created this PDF from an application that is not licensed to print to novaPDF printer (http://www.novapdf.com)

	64	42	57	66	85	65	67	61	74	25		
	66	30	52	56	63	59	57	59				
	72	41	59	58	64	60	56	55				
	69	25	49	60	81	64	63	66				
	61	36	56	75	95	88	73	66				
	66	25	38	41	80	57	53	24				
	62	51	75	65	88	70	74	64				
**Mean**	**67,31**	**36,31**	**56,85**	**60,08**	**79,31**	**67,15**	**64,85**	**59,15**	**71,43**	**37,71**	**42,60**	**38,25**
**SD**	**4,35**	**7,60**	**8,38**	**8,73**	**11,44**	**9,12**	**7,39**	**12,63**	**6,11**	**8,58**	**7,30**	**13,72**

HR

	S-Basal	S-Hemo15	S-Shock30	S-Shock60	S-R 0	S-R 60	S-R 120	S-R 180	NS-Basal	NS-Hemo15	NS-Shock30	NS-Shock60
	88	193	193	245	174	140	125	157	114	234	252	195
	117	199	231	236	185	162	164	159	120	270		
	112	252	273	183	165	154	159	285	93	185	247	261
	99	207	230	237	194	160	166	166	101	228	276	
	125	220	242	249	171	146	144	144	121	215	225	244
	82	181	184	175	119	112	120	113	90	191	208	245
	102	147	161	176	157	122	133	128	174	228		
	119	218	229	237	166	176	174	170				
	106	177	214	232	156	140	133	120				
	94	187	223	233	180	160	147	149				
	104	202	204	229	171	178	145	132				
	100	160	214	213	169	149	139	139				
	117	195	225	238	189	158	154	152				
**Mean**	**105,00**	**195,23**	**217,15**	**221,77**	**168,92**	**150,54**	**146,38**	**154,92**	**116,14**	**221,57**	**241,60**	**236,25**
**SD**	**12,73**	**26,97**	**27,93**	**26,39**	**18,87**	**18,99**	**16,45**	**42,75**	**28,40**	**28,57**	**26,10**	**28,58**

CVP

	S-Basal	S-Hemo15	S-Shock30	S-Shock60	S-R 0	S-R 60	S-R 120	S-R 180	NS-Basal	NS-Hemo15	NS-Shock30	NS-Shock60
	8	4	7	5	12	8	8	8	9	6	8	7
	8	3	5	5	11	8	7	7	9	6		
	8	6	7	12	10	9	9	7	12	4	6	5
	9	8	9	7	12	15	12	9	4	1	1	
	11	9	9	9	12	12	12	11	6	1	0	0
	8	3	4	5	13	9	8	7	8	2	2	3
	11	7	8	7	17	13	12	11	6	4		
	9	6	7	7	10	10	9	11				
	11	8	8	9	12	11	11	11				
	8	4	4	3	10	7	7	8				
	10	6	8	4	12	10	10	10				
	11	7	7	7	14	11	12	11				
	10	5	7	7	14	10	9	8				
**Mean**	**9,38**	**5,85**	**6,92**	**6,69**	**12,23**	**10,23**	**9,69**	**9,15**	**7,71**	**3,43**	**3,40**	**3,75**
**SD**	**1,33**	**1,95**	**1,66**	**2,39**	**1,96**	**2,20**	**1,93**	**1,72**	**2,63**	**2,15**	**3,44**	**2,99**

SVRI

	S-Basal	S-Hemo15	S-Shock30	S-Shock60	S-R 0	S-R 60	S-R 120	S-R 180	NS-Basal	NS-Hemo15	NS-Shock30	NS-Shock60
	1458	1827	1615	1923	862	1258	1361	1335	1512	1767	2082	
	1013	1516	1315	1374	479	786	654	711	1173			
	1061	1644	1929	1569	564	744	889	854	1666	2107	2549	2511
	1281	1019	1380	1305	527	856	905	876	1042	1568	1867	
	1243	1745	2011	1860	648	902	1003	1101	847	1062	1752	2044
	1658	2001	2063	2098	461	758	847	1109	992	1113	1579	1493
	923	1826	1529	1376	637	724	884	908	982	790		
	1005	1267	1340	1219	539	673	788	793				
	770	918	927	942	436	559	757	649				
	1055	1517	1816	1835	723	1047	981	998				
	939	1193	1523	1612	699	798	913	866				
	1198	1654	1668	1379	735	951	1095	1174				
	1017	1598	1890	2264	752	1043	976	893				
**Mean**	**1124,69**	**1517,31**	**1615,85**	**1596,62**	**620,15**	**853,77**	**927,15**	**943,62**	**1173,43**	**1401,17**	**1965,80**	**2016,00**
**SD**	**239,37**	**328,32**	**327,24**	**379,94**	**130,54**	**185,67**	**173,40**	**193,09**	**302,94**	**496,28**	**373,73**	**509,58**

PPV

	S-Basal	S-Hemo15	S-Shock30	S-Shock60	S-R 0	S-R 60	S-R 120	S-R 180	NS-Basal	NS-Hemo15	NS-Shock30	NS-Shock60
	7,3	14,5	14,5	30,5	12,1	17,3	21,7	15,4	8,1		24,9	
	13,4	25,7	30,8	31,2	17,9	19	16,8	20,4	9,2			
	7,7	22,6	16,7	12,4	9,7	13,8	16,1	15,8	8	16,3	12,8	
	7,5	32,3	35,5	36,2	13,9	14,6	14,9	16	12,1	34,2	20,6	18,8
	10,2	25,8	28,6	23,4	13,7	10,5	17,5	16,6	5	11,6	22,2	20,1
	7,9	28,2	30	28,6	7,9	9,1	8,7	15,1	4,8	24,8	27,6	15,1
	5,4	36,2	36,1	35,6	4,7	8,4	7,2	9,3	15,8	25,8		
	12,5	40,4	36	36,8	11,6	17	19,4	20,3				
	7,1	25,7	23,6	22,5	6,7	10,3	9,5	11,4				
	7,6	44,9	40,1	45,4	8	17,9	18,8	16,6				
	3,5	27,6	18,2	21,5	3,7	5,1	7	4,3				
	2,4	31,7	31,6	16,1	2	8,5	9,8	6,6				
	6,9	25,3	23,7	20,2	6,8	15,4	14,9	15,4				
**Mean**	**7,65**	**29,30**	**28,11**	**27,72**	**9,13**	**12,84**	**14,02**	**14,09**	**9,00**	**22,54**	**21,62**	**18,00**
**SD**	**3,08**	**7,91**	**8,17**	**9,40**	**4,56**	**4,43**	**4,99**	**4,87**	**3,90**	**8,81**	**5,61**	**2,59**

SvO2

	S-Basal	S-Hemo15	S-Shock30	S-Shock60	S-R 0	S-R 60	S-R 120	S-R 180	NS-Basal	NS-Hemo15	NS-Shock30	NS-Shock60
	72,3	43,7	53,3	49	73,7	66,5	64	62,8	76,1	29,2	21,8	9,8
	78,1	28,1	47	47,4	79,8	71,4	66,5	70,6	78,1	53,3		

You created this PDF from an application that is not licensed to print to novaPDF printer (http://www.novapdf.com)

	68,4	31	41,1	26,6	75,7	61,7	41,1	61,4	71,7	28,1	83	35,8
	75,6	91,6	48,1	50,1	78	68,8	71,3	68,4	75,3	46,4	38,1	
	77,1	35,1	31,7	27,8	71,9	53,5	60	66,3	80,1	55,6	28,2	25,8
	81,9	42,2	55,6	55,7	88,8	87,5	84,4	82	86,6	53,5	33,5	29,5
	91,5	74	74	73,8	75,9	79,4	74,4	78,6	85,8	25,7		
	82,2	40,5	62,5	69	81	80,5	79,4	76,2				
	84,4	64	73,3	69,8	82,2	74,3	73,9	75,8				
	78,9	25,7	43,8	50,6	76,9	62	60,2	62,1				
	76,7	39,8	61,8	52,3	74,4	67,7	57,9	61,6				
	80,2	28,7	40,8	49,1	80,5	77,3	70,4	71,2				
	77,1	45,9	59,4	50,8	78,2	67,5	67,3	69				
**Mean**	**78,80**	**45,41**	**53,26**	**51,69**	**78,23**	**70,62**	**66,98**	**69,69**	**79,10**	**41,69**	**40,92**	**25,23**
**SD**	**5,68**	**19,68**	**12,77**	**14,05**	**4,39**	**9,15**	**10,98**	**6,87**	**5,50**	**13,46**	**24,30**	**11,08**

Lactate

	S-Basal	S-Hemo15	S-Shock30	S-Shock60	S-R 0	S-R 60	S-R 120	S-R 180	NS-Basal	NS-Hemo15	NS-Shock30	NS-Shock60
	1,9	2,8	3,7	2,4	4,2	1,9	1,1	1	1,9	3,9	9,3	16,5
	1,7	4,5	4,5	4,1	8	2,2	1,3	1	1,5	5,9		
	2,4	3,6	3,4	5	6,9	2,3	1,4	1,3	2,1	4,6	6,1	6,2
	2,3	3,9	3,9	3,8	3,8	4,3	4,3	4,2	1,8	2,5	9,7	
	1,6	5,1	5,6	7	9	2,9	1,5	1,3	1,5	2,1	3,9	10,5
	1,2	1,5	2,1	2,4	7,5	3,3	2,1	1,5	2,3	3,2	7,1	13,4
	1,2	3	3	2,7	6,7	3	2	1,3	0,7	4,8		
	0,9	2,3	2,8	2,9	6,7	4	3	2,1				
	0,8	1	1,4	1,8	6	2,2	1,1	1				
	1,2	4,7	3,5	2,6	7	2,1	1,1	0,9				
	2,2	6	6	5,9	9	4,5	1,4	1,4				
	1,7	6,1	5,6	5,4	8,1	3,5	2	1,7				
	1,1	3,3	3	5,4	7,1	2,6	1,7	1,6				
**Mean**	**1,55**	**3,68**	**3,73**	**3,95**	**6,92**	**2,98**	**1,85**	**1,56**	**1,69**	**3,86**	**7,22**	**11,65**
**SD**	**0,53**	**1,59**	**1,38**	**1,64**	**1,57**	**0,88**	**0,91**	**0,86**	**0,52**	**1,35**	**2,39**	**4,38**

pH

	S-Basal	S-Hemo15	S-Shock30	S-Shock60	S-R 0	S-R 60	S-R 120	S-R 180	NS-Basal	NS-Hemo15	NS-Shock30	NS-Shock60
	7,42	7,38	7,34	7,34	7,39	7,44	7,44	7,44	7,43	7,37	7,27	7,16
	7,36	7,3	7,23	7,23	7,27	7,36	7,35	7,36	7,33	7,22		
	7,41	7,42	7,36	7,33	7,03	7,44	7,49	7,48	7,41	7,42	7,33	7,25
	7,39	7,38	7,35	7,35	7,35	7,44	7,41	7,47	7,417	7,369	7,23	
	7,41	7,4	7,32	7,29	7,18	7,43	7,43	7,41	7,399	7,436	7,381	7,252
	7,35	7,34	7,29	7,29	7,24	7,36	7,39	7,38	7,41	7,393	7,292	7,15
	7,5	7,42	7,42	7,39	7,32	7,44	7,4	7,43	7,35	7,3		
	7,47	7,48	7,39	7,36	7,34	7,42	7,42	7,44				
	7,38	7,34	7,32	7,35	7,32	7,36	7,42	7,41				
	7,39	7,33	7,32	7,32	7,27	7,39	7,41	7,41				
	7,41	7,41	7,32	7,32	7,26	7,33	7,43	7,42				
	7,45	7,43	7,38	7,39	7,37	7,44	7,43	7,53				
	7,42	7,48	7,41	7,38	7,26	7,41	7,43	7,44				
**Mean**	**7,41**	**7,39**	**7,34**	**7,33**	**7,28**	**7,40**	**7,42**	**7,43**	**7,39**	**7,36**	**7,30**	**7,20**
**SD**	**0,04**	**0,06**	**0,05**	**0,05**	**0,09**	**0,04**	**0,03**	**0,04**	**0,04**	**0,08**	**0,06**	**0,06**

BIC

	S-Basal	S-Hemo15	S-Shock30	S-Shock60	S-R 0	S-R 60	S-R 120	S-R 180	NS-Basal	NS-Hemo15	NS-Shock30	NS-Shock60
	27,5	24,5	22,3	24,2	26,8	28,8	28,4	28,2	25,6	20,9	16,9	8,2
	23,8	17,3	19,9	20,9	21,4	26,1	25	26,5	22,3	18,7		
	25,8	22,5	23,5	19,7	19,9	25,1	27,9	26,5	25,8	21,6	19,8	18,9
	25,9	25,2	24,7	24,2	23,5	27,1	24,5	27,1	25,4	19,3	13,8	
	27,3	20	20,7	18,2	18,9	24,2	24,8	22,4	24	21,2	20	13,8
	21,8	20,5	18,2	18,7	16,2	19,8	22,3	21,7	25,8	21,8	17,6	11
	28,2	26,4	26,4	22,9	21,8	25,7	24,9	25,3	16,3	11,5		
	26,4	21,9	21,2	19,4	19,3	23,9	24	25,1				
	22,3	20,3	21,2	19,6	19	23,4	23	23,6				
	26	18,6	22,9	23,6	21,1	25,9	26,2	27				
	26,4	22,4	23	22,3	20,2	24,4	28,9	29,3				
	26,5	17,4	20,6	20,6	20,9	24	21,9	27,7				
	25,2	24,9	24,7	22,7	20	25,3	26,6	26,6				
**Mean**	**25,62**	**21,68**	**22,25**	**21,31**	**20,69**	**24,90**	**25,26**	**25,92**	**23,60**	**19,29**	**17,62**	**12,98**
**SD**	**1,92**	**2,99**	**2,26**	**2,12**	**2,53**	**2,12**	**2,25**	**2,24**	**3,47**	**3,63**	**2,53**	**4,56**

K

	S-Basal	S-Hemo15	S-Shock30	S-Shock60	S-R 0	S-R 60	S-R 120	S-R 180	NS-Basal	NS-Hemo15	NS-Shock30	NS-Shock60
	4	4,5	4,2	4,3	4,1	4,6	4,5	4,8	3,7	4,1	5,4	7,1
	4,3	4	4,4	4,3	3,5	4,4	4,3	4,6	3,9	6,5		
	4	4,8	4,5	4,7	4,1	4,5	4,5	4,5	4,3	5,4	4,9	4,6
	3,9	4,1	3,9	3,8	3,8	4,3	4,3	4,2	3,7	4,1	6,3	
	3,9	6,2	4,3	4,8	3,7	4,4	4,7	4,7	4,1	4,1	4,7	6,1
	3,4	4	3,9	3,8	3,1	3,8	4,2	4,4	4,5	5	5,4	6,2
	3,9	4,1	4,1	3,7	3,9	4,2	4,4	4,1	4,6	8,4		
	3,6	5,7	3,6	3,4	4	4,3	4,3	4,2				
	3,8	3,9	3,9	3,9	4,2	4,8	4,6	4,5				
	3,9	5,1	4,8	5	4,7	4,6	4,5	4,7				
	3,7	6,7	4,4	4,6	3,9	4,4	5,4	5,5				
	3,8	5,9	4,5	4,6	4,4	4,2	4,5	4,1				
	4	6,5	4,2	5,1	4,2	4,8	4,8	4,6				
**Mean**	**3,86**	**5,04**	**4,21**	**4,31**	**3,97**	**4,41**	**4,54**	**4,53**	**4,11**	**5,37**	**5,34**	**6,00**
**SD**	**0,22**	**1,04**	**0,33**	**0,54**	**0,40**	**0,27**	**0,31**	**0,38**	**0,37**	**1,60**	**0,62**	**1,04**

You created this PDF from an application that is not licensed to print to novaPDF printer (http://www.novapdf.com)

DeltaCO2

	S-Basal	S-Hemo15	S-Shock30	S-Shock60	S-R 0	S-R 60	S-R 120	S-R 180	NS-Basal	NS-Hemo15	NS-Shock30	NS-Shock60
	10,8	16,6		15,1	8,6	9,6	11,4	9	10,1	14,8	32,3	57,3
	10,4	33,4	24,3	19,7	7,5	7,2	8,5	9,7	8,4	0,7		
	12,5	27,1	22,4	25,7	9	9,9	8,4	11	9,4	23,5	30,5	23
	12,1	21,5	17,9	15,6	8,6	8,2	8,5	8,1	9	19,9	35,6	
	13	32,8	27,3	33,2	9,2	14,5	13,1	15,7	6,3	14,1	18,9	28,1
	9,1	17,8	19,9	16,6	5,5	4,9	3,2	7,9	12,9	19,8	18,4	22,8
	5	6,7	6,7	11,8	4,2	7,3	5,8	6,2	15,7	30		
	6,3	16,9	13	16,6	5,6	4	5,9	5,9				
	10,1	14,2	14,4	14,3	4,2	4,8	6,7	7,4				
	4,5	25,2	18,3	16,5	7,2	10,8	11,5	12,4				
	9,2	21,9	13,1	16,7	7,5	10	13,9	9,3				
	7	34,3	18,4	19,3	6,8	9,3	10,8	8,2				
	12	21,6	15,8	18,4	7,2	10,7	10,1	9,7				
**Mean**	**9,38**	**22,31**	**17,63**	**18,42**	**7,01**	**8,55**	**9,06**	**9,27**	**10,26**	**17,54**	**27,14**	**32,80**
**SD**	**2,87**	**8,17**	**5,58**	**5,53**	**1,69**	**2,92**	**3,12**	**2,63**	**3,11**	**9,17**	**7,97**	**16,52**

You created this PDF from an application that is not licensed to print to novaPDF printer (http://www.novapdf.com)
